# Does accelerometer-measured physical activity and sedentary time differ between manual, in-office, hybrid and remote workers?

**DOI:** 10.1136/oemed-2025-110105

**Published:** 2025-06-25

**Authors:** Tuija Leskinen, Kristin Suorsa, Jesse Pasanen, Suvi Rovio, Harri Niinikoski, Olli Heinonen, Laura Pulkki-Råback, Jorma Viikari, Tapani Rönnemaa, Olli T Raitakari, Sari Stenholm, Katja Pahkala

**Affiliations:** 1 Department of Public Health, University of Turku, Turku, Finland; 2 Centre for Population Health Research, University of Turku, Turku, Finland; 3 Research Centre of Applied and Preventive Cardiovascular Medicine, University of Turku, Turku, Finland; 4 Department of Pediatrics and Adolescent Medicine, University of Turku, Turku, Finland; 5 Paavo Nurmi Centre and Unit for Health and Physical Activity, University of Turku, Turku, Finland; 6 Department of Psychology, University of Helsinki, Helsinki, Finland; 7 Department of Medicine, University of Turku, Turku, Finland; 8 Division of Medicine, TYKS Turku University Hospital, Turku, Finland; 9 Department of Clinical Physiology and Nuclear Medicine, TYKS Turku University Hospital, Turku, Finland; 10 Research Services, TYKS Turku University Hospital, Turku, Finland

**Keywords:** Physical Activity, Workers, Epidemiology, Public health

## Abstract

**Objectives:**

Studies on accelerometer-measured daily physical activity behaviour, especially among hybrid and remote workers, are scarce. We compared daily occupational and non-occupational physical activity and sedentary time among manual, in-office, hybrid and remote workers. In addition, physical activity behaviour during remote and office workdays among hybrid workers was compared.

**Methods:**

Daily physical activity behaviour was collected with wrist-worn accelerometers on ≥4 days from 133 Finnish workers (31 years, 61% women). Participants were divided into four groups according to their work modes: manual (n=32), in-office (n=49), hybrid (n=35) and remote workers (n=17). Differences in physical activity and sedentary time during workdays (separately for occupational and non-occupational time) between the groups were examined using generalised linear models. Linear mixed models were used for intra-individual differences among hybrid workers.

**Results:**

Workdays’ occupational physical activity and sedentary time differed between the work mode groups (p<0.0001); the manual workers accumulated the highest occupational physical activity, while both hybrid and remote workers accumulated the highest occupational sedentary time. No differences in non-occupational behaviours were observed. Among hybrid workers, occupational sedentary time tended to be higher (26 min, 95% CI −2 to 53) during remote versus office workdays, but non-occupational behaviours were similar.

**Conclusions:**

Remote work is associated with the lowest physical activity and the highest sedentary time compared with other work modes. Strategies to promote physical activity during remote workdays may be needed.

**WHAT IS ALREADY KNOWN ON THIS TOPIC**

- Manual workers are physically active during working hours, whereas non-manual workers are mostly sedentary at their work. Remote work is associated with high sedentary time.

**WHAT THIS STUDY ADDS**

- On workdays, both hybrid and remote workers accumulated less occupational physical activity and more occupational sedentary time than in-office or manual workers. Hybrid workers tend to accumulate lower occupational physical activity during remote compared with office workdays.

**HOW THIS STUDY MIGHT AFFECT RESEARCH, PRACTICE OR POLICY**

- Tailored recommendations and new interventions to increase daily physical activity during remote workdays are warranted.

## Introduction

 Health-enhancing physical activity can accumulate during worktime, domestic chores (eg, housework, yard work, shopping), leisure time or transportation.^1^ Hence, in terms of aiming to increase physical activity for health, it is important to study physical activity within all these different domains. However, questionnaire-based assessments have mostly been limited to one domain at a time, whereas accelerometer-based measurements provide a more accurate measure of the accumulation of physical activity during the whole day.^1 2^

It is known that physical activity at work depends markedly on one’s occupation.^3^ Previous accelerometer-based studies have shown that manual workers accumulate the majority of their daily physical activity during the working hours,^4^ while non-manual workers are sedentary most of their worktime.^3^ These patterns have raised concerns related to their associations with different health outcomes.^5^ For example, it is not known whether occupational physical activity provides similar health benefits as leisure time physical activity.^6^ In addition, high or prolonged sedentary time has been linked to higher levels of cardiometabolic risk factors,^7^ musculoskeletal pain^5 8^ as well as a higher risk for cardiovascular diseases and mortality.^5 9 10^ On the other hand, interrupting long periods of sitting has been shown to offer health benefits.^7^ Therefore, the recommendations for daily physical activity among adults have been suggested to cover both occupational and non-occupational time.^5 11^ However, there is a need to understand in detail how occupational and non-occupational physical activity and sedentary behaviour accrue during the day among manual and non-manual workers.^11^

Five years ago, the working life changed dramatically when the COVID-19 pandemic led to an unprecedented growth of remote work. Subsequently, for example, half of the office workers in Europe^12^ continued work remotely or with a hybrid model of working, that is, a combination of remote and in-office work. In the pre-pandemic survey studies, remote workers have reported lower physical activity levels than in-office workers because of a lack of active commuting,^13^ longer working hours^14^ and increased work-related sitting.^15^ More recent survey-based findings suggest that remote work reduces daily physical activity.^16^ However, accelerometer-based studies on daily physical activity and sedentary behaviour among remote workers are needed as the aforementioned findings may be affected by the recall and information bias linked to self-reported data^1^ as well as the pandemic-related restrictions per se.^17^ Also, the recent concern of high sedentary time among remote workers warrants detailed investigations of the occupational physical activity behaviour during remote working hours so that future interventions and actions can be better applied to remote environments.^18^

To fill the gap in accelerometer-based studies,^18^ this study aimed to examine daily physical activity and sedentary time during workdays (separately for occupational and non-occupational time) and days off in various modes of work. These work modes included manual workers (ie, work requiring completion of physical tasks), in-office workers (work carried out in an office environment), hybrid workers (work carried out from the office and remotely) and remote workers (work done remotely). Moreover, we aimed to compare occupational and non-occupational physical activity and sedentary time during remote and office workdays among the hybrid workers.

## Methods

### Participants

This study used physical activity data collection nested to the follow-up of the Special Turku Coronary Risk Factor Intervention Project (STRIP) cohort.^19 20^ Overall, 396 individuals, who successfully wore the accelerometers during the STRIP follow-up at the age of 26 years^19^ were invited to a 1 week-long accelerometer measurement again at the age of 31 years. In total, 223 participants (56% of the invited) agreed to participate. Compared with those declining from the accelerometer measurement at the age of 31 (n=173), those who agreed were more frequently women (64% vs 50%, p<0.01), reported to work (70% vs 60%, p=0.07) and tended to have slightly lower body mass index (24.1 vs 24.9 kg/m^2^, p=0.08) at the age of 26 years.

Overall, 217 participants lived in Finland and received accelerometers via mail. Of those, 203 wore the accelerometer and provided accelerometer data for at least four valid measurement days. For the analysis of the present study, we included those 133 participants (81 women, 52 men) who reported to work either full-time or part-time, had reported their current job title, and had at least one valid workday measurement day. The accelerometer measurements were conducted between May 2021 and May 2022, during a time period of partial lockdown and recommendations for remote work in Finland.

### Physical activity measurement

Physical activity was measured over seven consecutive days and nights using triaxial ActiGraph wActiSleep-BT accelerometers and the newer wGT3X-BT accelerometers (ActiGraph, Pensacola, Florida, USA), replacing the broken devices. The accelerometer was initialised to record at 80 Hz. Participants were instructed to wear the device on their non-dominant wrist at all times, including during water-based activities such as swimming, but to remove it for the sauna. Data from the accelerometers were downloaded and converted into 60 s epochs in ActiLife software, V.6.13 (ActiGraph, Pensacola, Florida, USA). Vector magnitude (VM) counts per minute (CPM) were calculated as the square root of the sum of squared activity counts of the three axes. The participants were given participants’ logs in which they recorded information about each measurement day, including waking and sleeping times, workdays and days off, work times, as well as whether or not they had worked remotely on that particular day.

We included wear time between the first and last recorded time in the participant log, and excluded non-wear time using the algorithm developed by Choi and sleep time by the algorithm available in the ActiLife software.^19^ A valid measurement day was defined as a minimum of 10 hours of wake accelerometer wear time. A minute-by-minute data were produced for all valid measurement days. A minute was defined as active when the VM of the wrist-worn accelerometer equalled or exceeded 1853 CPM; a sedentary minute was defined as less than 1853 CPM, as validated against thigh-worn accelerometers.^21^ The mean sums of active and sedentary minutes were calculated for all days, and separately for workdays and days off, and for workday occupational and non-occupational time. Occupational time was defined as accelerometer wear time during reported working hours. Respectively, non-occupational time was defined as accelerometer wear time outside the reported working hours, for example, time spent in transportation, leisure and household activities. The mean activity minutes for each hour of the workday were calculated to illustrate the daily physical activity profiles during typical waking hours (from 06:00 to 23:00) for each work mode group.

Among hybrid workers, activity and sedentary bouts, that is, periods of continuous physical activity or sedentary time, were calculated from the minute-by-minute activity data for each work shift. The length of the activity or sedentary bout was defined as the number of consecutive minutes spent in an active or sedentary state, respectively. The sum of time spent in long sedentary bouts (>30 min) was calculated for the working hours of each workday. A fragmentation index was calculated as the reciprocal of the average bout length for both physical activity and sedentary behaviour. The fragmentation index is used as a marker of fragmented behaviour and expressed as the probability of transitioning from active to sedentary state (or vice versa for sedentary fragmentation index).^22 23^

### Modes of work

The occupation-based social classification was done from the self-reported job titles using the Goldthorpe class scheme.^24^ Participants were first dichotomised into non-manual and manual workers. The non-manual workers were those having Goldthorpe classes I (including higher grade professionals, administrators and officials), II (lower grade professionals, administrators and officials), III (routine non-manual employees), IV (small proprietors) and V (lower grade technicians, supervisors of manual workers). The manual workers had Goldthorpe classes VI (skilled manual workers), VIIa (semi-skilled and unskilled manual workers) and VIIb (agricultural workers, other workers in primary production). Second, four groups, each referring to a specific mode of work, were created according to the participants’ occupational status and their log data of workdays’ modes during the measurement week. The four groups were: manual workers (n=32), in-office workers (non-manual workers who had only office workdays, n=49), hybrid workers (non-manual workers who had both remote and office workdays, n=35) and remote workers (non-manual workers who had only remote workdays, n=17).

### Covariates

Sex, employment status (full-time vs part-time), level of education (basic, ie, at least upper secondary education or currently studying vs advanced, ie, education obtained from university or university of applied sciences, whether ongoing or completed), marital status (single vs married or cohabiting) and current health status (good, ie, very good or pretty good vs sub-optimal, ie, average, fair or poor perceived health) as well as work-time mode (regular day work vs shift work, that is, two-shift, three-shift, night shift and irregular worktimes) were assessed with the web-based questionnaire at the time of the accelerometer measurements.

### Statistical analyses

The characteristics of the participants by work mode groups are presented as mean values and SD for the continuous variables, and as numbers and percentages for the categorical variables. Differences between the work mode groups were examined using χ^2^ test for categorical variables and analysis of variance for continuous variables.

The differences in physical activity and sedentary time between the work mode groups were examined using generalised linear models, adjusted for sex, education, employment status, marital status, perceived health, shift work and accelerometer wear time (daily, workday, day off, occupational and non-occupational wear time depending on the analysis). The intra-individual differences between remote and office workdays among the hybrid workers were examined using linear mixed models, adjusted for accelerometer wear time (daily, occupational and non-occupational wear time depending on the analysis). The results are provided as mean estimates and mean difference estimates and their 95% CIs. The SAS Software V.9.4 was used for the statistical analyses (SAS Institute, Cary, North Carolina).

## Results

Characteristics of all participants and by the work mode groups are provided in table 1. All participants were Caucasian and aged 31 (SD 0) years at the time of the accelerometer measurements. Overall, 61% of the participants were women, 71% had advanced education, 88% were working full-time, 77% were married or cohabiting, 77% had regular day work and 64% had good self-reported health. Manual workers were less likely to have advanced education compared with other work mode groups. One-third of the manual and office workers were shift workers. Hybrid workers were more likely to have part-time work than other workers in other work modes. The participants provided an average of 6.8 (SD 0.5, range 5–8) valid measurement days. There were no statistical differences in the mean accelerometer wear time (p=0.24) nor in the mean work times (p=0.44) across the work mode groups (table 1).

**Table 1:** Characteristics of the participants by the work mode groups

Characteristics	All	Manual workers	Non-manual workers			P for model*
			In-office workers	Hybrid workers	Remote workers	
N	133	32	49	35	17	
Age, years	31 (SD 0)	31 (SD 0)	31 (SD 0)	31 (SD 0)	31 (SD 0)	
Women, n (%)	81 (61)	17 (53)	30 (61)	23 (66)	11 (65)	0.74
Advanced education, n (%)	95 (71)	5 (16)	42 (86)	31 (89)	17 (100)	<0.0001
Working full-time, n (%)	117 (88)	28 (88)	47 (96)	26 (74)	16 (94)	0.02
Married/cohabiting, n (%)	101 (77)	20 (63)	41 (86)	27 (79)	12 (71)	0.10
Good perceived health, n (%)	85 (64)	15 (47)	36 (73)	23 (66)	11 (65)	0.11
Regular day workers, n (%)	102 (77)	22 (69)	32 (65)	31 (89)	17 (100)	0.006
Valid measurement days, n (95% CI)	6.8 (SD 0.5)	6.8 (6.6 to 6.9)	6.9 (6.8 to 7.0)	6.9 (6.7 to 7.0)	6.8 (6.5 to 7.0)	0.62†
Mean accelerometer wear time, min/day (95% CI)	966 (SD 53)	979 (961 to 998)	966 (951 to 981)	953 (935 to 970)	966 (941 to 991)	0.24†
Mean worktime‡, min/workday (95% CI)	481 (SD 78)	492 (463 to 521)	482 (457 to 506)	484 (457 to 511)	452 (413 to 491)	0.44†

* For the comparison between the four work mode groups using χ2 test.

† P value from the generalised linear model.

‡ Accelerometer wear time during reported working hours.

### Comparisons between the work mode groups

Daily physical activity and sedentary time for all days, workdays and days off for each work mode group are given in table 2. Manual workers were more physically active than the in-office, hybrid and remote workers on all days (p=0.0005) and on workdays (p<0.0001). On workdays, occupational physical activity and sedentary time differed between the work mode groups (p<0.0001), whereas non-occupational behaviours were similar (p=0.44). The manual workers had higher occupational physical activity (259 min) compared with in-office (193 min), hybrid (137 min) and remote (143 min) workers. In terms of sedentary behaviour, on workdays, the manual workers had 1–2 hours less occupational sedentary time compared with all the non-manual work mode groups. Also, the in-office workers accumulated 1 hour less occupational sedentary time compared with the hybrid and remote workers. Workdays’ physical activity and sedentary behaviour did not differ between the hybrid and remote workers. Also, no differences for physical activity or sedentary time during days off were observed across the work mode groups (p=0.08), although the remote workers showed the highest sedentary time also during days off (table 2).

**Table 2:** Daily physical activity and sedentary time for all days, workdays and days off by the work mode groups

	Manual workers	Non-manual workers			P for model*
	Mean minutes (95% CI)	In-office workers	Hybrid workers	Remote workers	
		Mean minutes (95% CI)	Mean minutes (95% CI)	Mean minutes (95% CI)	
Physical activity (PA)					
Daily total PA (all days)	445 (389 to 501)	387 (330 to 444)†	347 (290 to 405)†‡	324 (259 to 389)†‡	0.0005
Workday’s total PA	478 (417 to 540)	412 (349 to 475)†	346 (283 to 409)†‡	330 (259 to 400)†‡	<0.0001
Workday’s occupational PA	259 (203 to 314)	193 (137 to 249)†	137 (81 to 194)†‡	143 (79 to 207)†‡	<0.0001
Workday’s non-occupational PA	218 (177 to 259)	216 (173 to 259)	202 (159 to 245)	189 (140 to 238)	0.44
Days off total PA	392 (332 to 453)	352 (290 to 414)	354 (292 to 416)	299 (227 to 372)†	0.08
Sedentary time (SED)					
Daily total SED (all days)	521 (465 to 577)	579 (522 to 636)†	519 (561 to 676)†‡	642 (577 to 707)†‡	0.0005
Workday’s total SED	518 (457 to 579)	584 (521 to 646)†	650 (588 to 712)†‡	666 (596 to 712)†‡	<0.0001
Workday’s occupational SED	222 (166 to 278)	288 (232 to 343)†	343 (287 to 399)†‡	337 (273 to 401)†‡	<0.0001
Workday’s non-occupational SED	371 (330 to 412)	372 (330 to 415)	387 (344 to 429)	400 (351 to 449)	0.44
Days off total SED	507 (447 to 568)	548 (485 to 568)	546 (484 to 608)	600 (528 to 672)†	0.08

All models adjusted for sex, education, employment status, marital status, perceived health, shift work and accelerometer wear time.

* For the comparison between the four work mode groups.

† Significant difference compared with manual workers.

‡ Significant difference compared with in-office workers.

**Figure 1:**
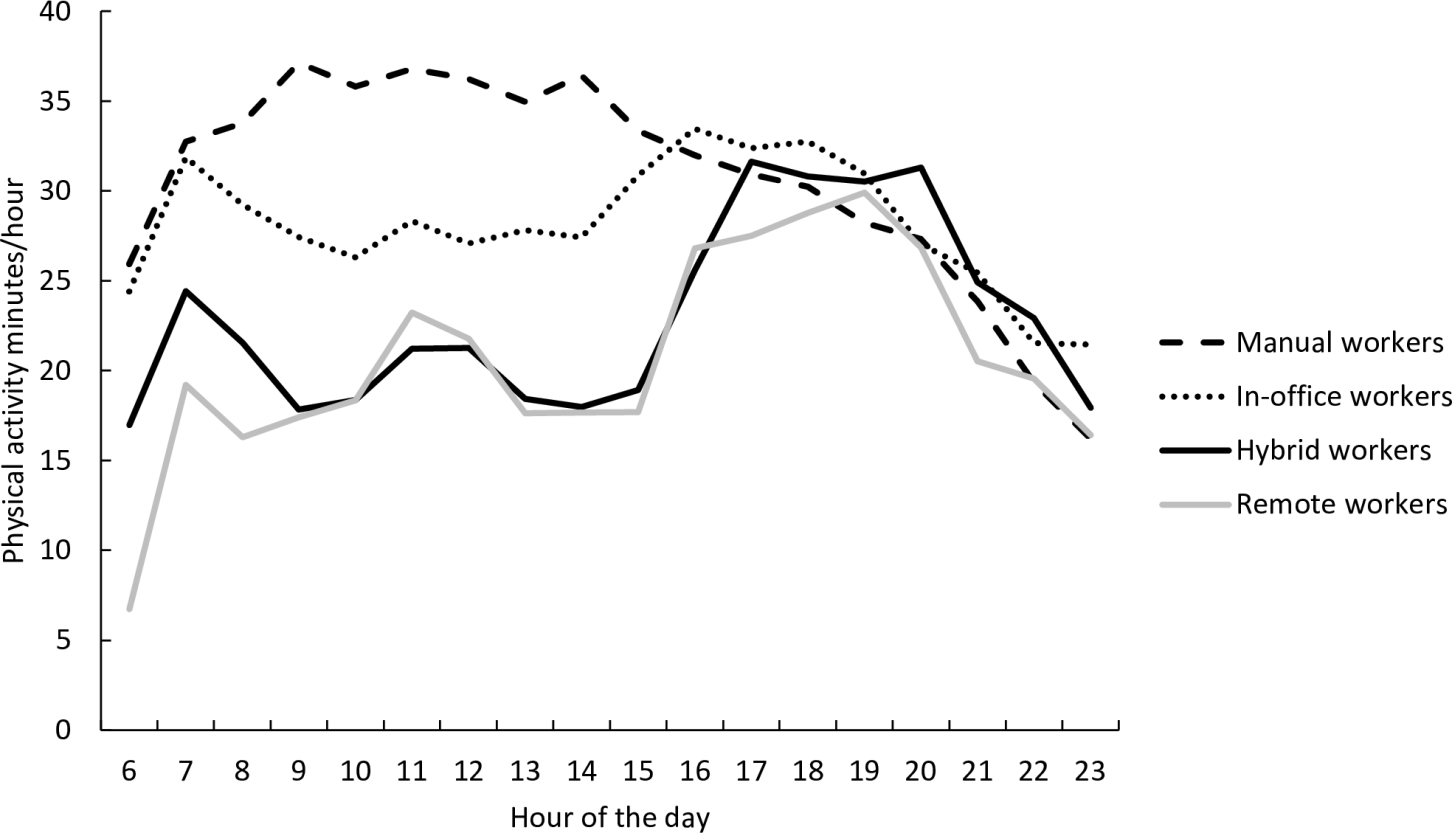
Descriptive physical activity profiles of an average workday for manual, in-office, hybrid and remote workers.

Figure 1 illustrates the wake-time physical activity profiles for an average workday for each work mode group. The manual workers seemed to accumulate high physical activity during the morning hours, whereas the in-office, hybrid and remote workers increased their physical activity more during the afternoon hours. Compared with the in-office workers, who commute and work at the office every workday, both hybrid and remote workers accumulated less physical activity during their workdays and did not reach the activity level of the in-office workers until the late evening hours. When excluding the shift workers (n=15) and persons with irregular work times (n=16), the daily profiles did not markedly change, but emphasised the high physical activity among the manual workers during the typical working hours (online supplemental figure 1).

### Remote versus office workdays among hybrid workers

**Figure 2:**
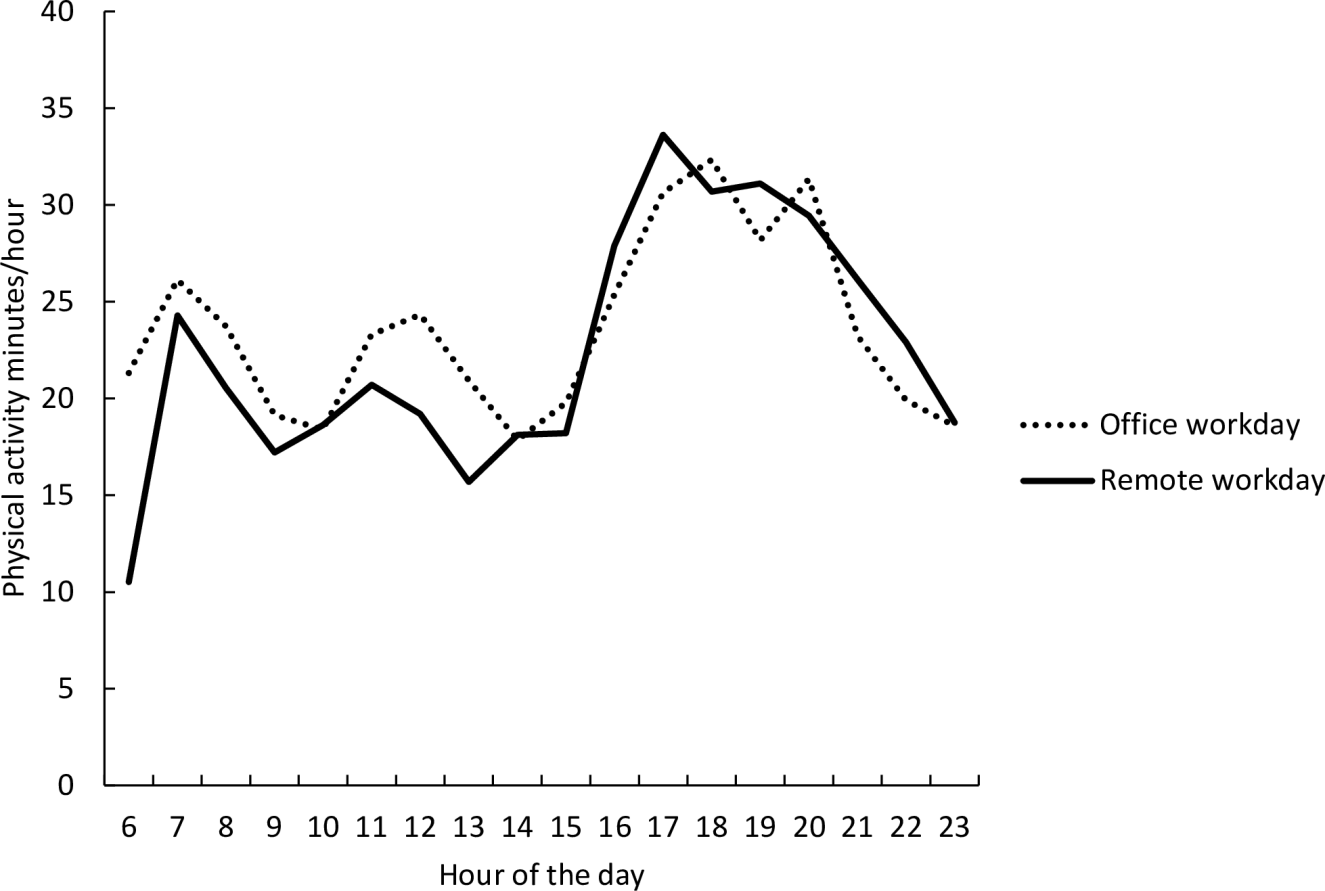
Descriptive physical activity profiles for office and remote workdays among hybrid workers.

Among the hybrid workers, within-person analysis was conducted by comparing daily total, occupational and non-occupational physical activity and sedentary time for office versus remote workdays (table 3). Compared with the office days, the remote workdays accumulated less physical activity and more sedentary time, especially during worktime, although the differences only approached significance. The occupational physical activity tended to be lower for remote compared with office workdays (mean difference −26 min, 95% CI −53 to 2, p=0.06), with a significantly higher activity fragmentation, that is, the probability for transitioning from active to sedentary state during the remote working hours (42% vs 36%, p=0.04). Sedentary behaviour tended to be half an hour higher during remote workdays, but no differences, for example, in the sum of time spent in long sedentary bouts were seen. Also, the non-occupational behaviours did not differ between the office and remote workdays (table 3). Figure 2 illustrates the rather similar physical activity patterns for the remote and office workdays among the hybrid workers.

**Table 3:** Daily total, occupational and non-occupational physical activity and sedentary time for office and remote workdays among hybrid workers

	Office workday	Remote workday	Difference
	Mean minutes (95% CI)	Mean minutes (95% CI)	Mean minutes (95% CI)
Worktime	487 (460 to 514)	463 (437 to 490)	−24 (−59 to 12)
Physical activity (PA)			
Daily total PA	389 (355 to 424)	362 (328 to 397)	−27 (−54 to 1)
Occupational PA	160 (138 to 182)	135 (113 to 157)	−26 (−53 to 2)
Worktime activity bout length	3.4 (2.7 to 4.1)	2.6 (1.9 to 3.3)	−0.8 (−1.9 to 0.2)
Worktime activity bout number (n)*	51.9 (46.9 to 57.0)	51.7 (46.6 to 56.8)	−0.2 (−5.2 to 4.8)
Worktime PA fragmentation index†	0.36 (0.32 to 0.40)	0.42 (0.38 to 0.46)	0.06 (0.002 to 0.11)
Non-occupational PA	245 (219 to 271)	247 (221 to 273)	2 (−19 to 23)
Sedentary time (SED)			
Daily total SED	594 (560 to 629)	624 (589 to 658)	30 (−0.4 to 59)
Occupational SED	315 (293 to 337)	341 (319 to 362)	26 (−1.7 to 53)
Worktime sedentary bout length	7.0 (5.4 to 8.5)	7.4 (5.9 to 9.0)	0.4 (−0.9 to 1.8)
Worktime SED fragmentation index‡	0.19 (0.16 to 0.22)	0.16 (0.13 to 0.19)	−0.03 (−0.08 to 0.008)
Worktime long sedentary bouts sum§	84 (57 to 110)	85 (59 to 111)	2 (−29 to 32)
Non-occupational SED	323 (297 to 349)	321 (295 to 347)	−2 (−23 to 19)

All models adjusted for accelerometer wear time.

* Similar number for sedentary bouts.

† Probability of transitioning from active to sedentary state.

‡ Probability of transitioning from sedentary to active state.

§ Sum of time spent in long sedentary bouts.

## Discussion

We compared daily physical activity and sedentary time on workdays and days off among Finnish manual, in-office, hybrid and remote workers. We found that both the hybrid and remote workers were physically less active and had 1–2 hours higher occupational sedentary time compared with the in-office and manual workers. Furthermore, the within-person comparisons among hybrid workers suggested 26 min higher occupational sedentary time during remote versus office workdays.

Overall, our findings underline that occupational physical activity behaviour differs more than free time behaviour (ie, physical activity during non-occupational time and days off) between manual, in-office, hybrid and remote workers. The highest occupational physical activity among the manual workers complements the existing findings.^3 5^ However, because prior research shows that high occupational physical activity may not provide similar health benefits as leisure-time physical activity,^6 25^ it is important to both provide ways to lower the burden of physical work at workplaces (eg, with breaks and ergonomics) as well as to promote fitness-enhancing leisure-time physical activity among the manual workers.^5 11^

The workday and occupational physical activity were found to be the lowest among both the hybrid and remote workers as they accumulated 1–2 hours less physical activity during the working hours compared with the in-office and manual workers. This finding adds to previous survey-based studies, both before^13 15 26^ and during^27 28^ the pandemic showing lower physical activity and higher sedentary time during remote versus office workdays. Thus, our finding further emphasises the sedentary nature of remote work.^18^

Our more detailed analysis of the within-person differences between remote and office workdays among hybrid workers suggested lower occupational physical activity and 26 min higher occupational sedentary time during the remote working hours. This finding complements the more recent studies which have found higher daily sedentary time when working at home versus office.^29 30^ In line with our finding of the higher activity fragmentation index during remote working hours (ie, shorter activity bouts resulting a higher probability for transitioning from active to sedentary state), one study has previously found shorter activity bouts (<5 min) when working from home versus at the office.^30^ These findings suggest that occupational physical activity may be more fragmented during remote versus office work. This may be due to shorter transitions to, for example, lunch or coffee when working remotely (usually at home). However, although these short activity bouts are efficient for breaking up prolonged sitting,^7 31 32^ they may not be enough to reduce total sedentary time during remote workdays.

There is, thus, accumulating evidence that remote work is characterised by high sedentary time, which may elicit negative health consequences, especially for cardiometabolic health.^7 32^ Although multi-interventions have shown to be effective in reducing sedentary behaviour in office environments, on average by 40 min per day,^33^ the effectiveness of these interventions has not yet been studied in remote environments.^18^ Therefore, intervention studies aiming to increase daily physical activity and/or reduce sedentary time during remote workdays are warranted. Applicable ways to reduce sitting behaviour in office or remote environments are to use alerts or other reminders for breaking up sitting time and/or to increase physical activity, for example, via task-related transitions.^34^ Also, support and acceptance by the co-workers and managers for (longer) breaks from sitting when working remotely may be important,^35^ as the social environment has been shown to facilitate sitting reduction at the office.^36^

On the other hand, remote work has also been suggested to liberate more time for non-occupational physical activity because of reduced commute time, more flexible working hours and increased possibility to break up work with physical activities.^30 37^ However, it seems that this opportunity is not (yet) well used. For example, a study conducted during the pandemic showed that people slept longer in the morning on remote workdays,^38^ which could also explain the low physical activity levels during the morning hours among the hybrid and remote workers in our daily profiles. Also, a previous study among office workers found that the daily moderate-to-vigorous physical activity was lower when the work was done remotely versus at the office.^30^ One explanation for the finding may be the lack of active commuting, which in fact has been shown to be a crucial part of health-enhancing physical activity among the office workers.^5 39 40^ Unfortunately, we could not separate active commuting time in our study. Overall, these findings suggest that efficient ways to compensate for high sedentary time during remote work could be to break up sitting with physical activity, to have longer active breaks and/or to increase non-occupational physical activity during remote workdays.

This study yielded new scientific information on accelerometer-measured physical activity and sedentary behaviour among different work mode groups, including for the first time also hybrid and remote workers. With detailed participants’ logs, we were able to identify daily working hours to study both occupational and non-occupational physical activity and sedentary behaviour. However, our measurements were conducted during a period of partial lockdown and recommendations for remote work, which may have affected the findings. Thus, post-pandemic studies are needed to provide more up-to-date data on daily physical activity behaviour among especially hybrid and remote workers. In addition, our findings were derived from a rather small group of healthy and relatively young workers. Therefore, the findings need to be interpreted with caution and replicated with larger-scale studies, including wider representation of the working-aged adults. Finally, the known limitation related to wrist-worn accelerometers’ ability to measure sedentary time and certain types of physical activity (eg, cycling) should be acknowledged.^2^

### Conclusions

Compared with manual and in-office work, which are more traditional work modes, hybrid and remote work modes were characterised by lower physical activity and higher sedentary time. Therefore, updated recommendations and targeted interventions may be needed to reduce sedentary behaviour among current hybrid and remote workers.

## Supplementary material

10.1136/oemed-2025-110105 online supplemental file 1

## Data Availability

Data are available upon reasonable request.
