# Supplementary material for: Does accelerometer-measured physical activity and sedentary time differ between manual, in-office, hybrid and remote workers?
Source: Occup Environ Med. 2025 Jun 25;82(5):e110105. doi: 10.1136/oemed-2025-110105 (PMC12322443; doi:10.1136/oemed-2025-110105)

Supplementary Figure 1. Descriptive physical activity profiles of an average workday among regular day workers.

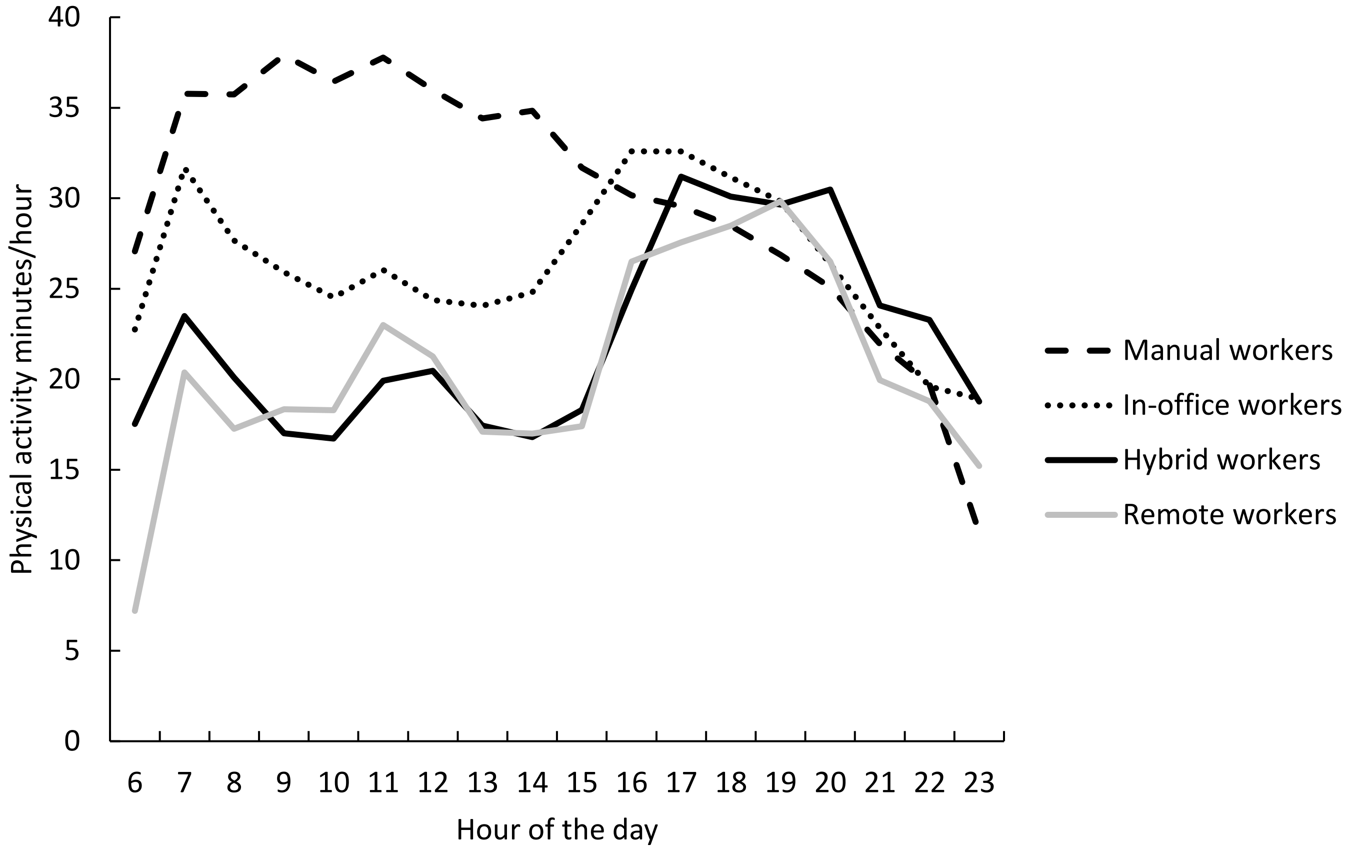

Supplement: online supplemental file 1 [file oemed-82-5-s001.pdf]
